# A Roll of Honor

**Published:** 1871-02

**Authors:** 


### A ROLL OF HONOR.

| | |
|---|---|
| W. W. Atterbury. | David Hoadley. |
| Nathan Bishop. | J. M. Morrison. |
| Wm. A. Booth. | John E. Parsons. |
| James W. Beekman. | Gustav Schwab. |
| Robert Carter. | Philip Schaff. |
| Thomas C. Doremus. | Otis D. Swan. |
| John Elliott. | William A. Smith. |
| Fred G. Foster. | William Walker. |
| John C. Havemeyer. | F. S. Winston. |

The names above are honored all over New York City, are repeated with deference, and remembered with respect always. They all stand high as citizens, socially and financially; they are foremost among our merchants, our bankers, and our business men; every one of them a leader in his line. They have banded together, and represent a committee which has been working, and working hard, for twelve years, quietly, efficiently, and with a dignity and self-respect worthy of all praise. They have been fighting in a manly manner and with manly weapons against one of the customs of the times, advocated and defended by large masses of men, representing millions and millions of dollars, and sustained by the learning, and the power, and the prestige of a part of the public press, which ought to have been found sustaining a nobler cause. But this committee have so used the weapons of their warfare, that while their opponents have literally gnashed their teeth in the rage of successive defeats, they command their highest respect, and dare not and cannot say aught against them.

This committee have freely spent their time and money for these successive years, and have used their influence and their energy with a steadiness of purpose, a singleness of aim, and an efficiency of action which merit the praise of all good men. In fair weather and foul, in victory or defeat, they have manifested the same calm, confident demeanor, always relying on the right of their cause, and the wide-reaching influences for good which its success would have in the elevation of the masses and the general weal of all, not only for the present, but in the ages to come.

This committee began twelve years ago to work for the better observance of the Sabbath day, first and mainly, in New York City. They believed that a properly observed Sabbath was not only the glory of any nation, but was its salvation, as well as its happiness; that it would mitigate many of the evils of ignorance and poverty; that it would diminish drunkenness, robbery, crime, and theft, besides saving much of human life and blood. Said a gentleman at Newport last year, in a public address, having spent a large portion of his life abroad, and comparing the Continental method of observing the Sabbath day with that of our own happy country, "If our Sabbaths are lost, all is lost." A little Sunday-school girl, who went abroad two years ago and returned recently, exclaimed the first Sunday on her return, "It's so nice to get back where there is a Sunday." This committee have issued, on an average, two reports every year, which, if purchased and bound together in one volume would contain an amount of Sabbath literature which would enrich any library; and such a volume ought to be placed in our principal public libraries for reference in after years, and where the names of the committee may be read with pleasurable pride by their descendants a hundred years to come, similar to that which fills the hearts now of the children's children of those who signed the Declaration of Independence. Letters and orders for these documents may be addressed to "The Secretary of the Sabbath Committee, No. 5 Bible House, New York." Every intelligent Sabbath-revering and Sabbath-loving man would do a public good, whether he lives in New York or not, by sending a donation, be it large or small, to J. M. Morrison, Esq., President of the Manhattan Bank, 40 Wall Street, New York. A burning shame will it be to allow this noble band of men to do all the work and bear all the expenses of it, — expenses of office rent, of postage, of printing, of going to Albany to the neglect of their business, spending days and weeks in privately presenting facts to the members of the Legislature, and urging them, by arguments drawn from two worlds, to give their influence to the proper observance of the blessed Sabbath day, the distinctive glory of the American and British people. Among the subjects treated by this committee are, First, The object and policy of the Committee. Second, Labors and results. Third, Metropolitan excise law. Fourth, Sunday processions. Fifth, Sunday railroad and post-office work. Sixth, Sabbath observance in other States, in Canada, Great Britain, on the Continent, etc.

